# The Eruption of the Teeth

**Published:** 1901-03-15

**Authors:** T. E. Constant

**Affiliations:** Scarborough, Eng.


### THE ERUPTION OF THE TEETH.

BY T. E. CONSTANT, SCARBOROUGH, ENG.

Being an extract from a paper read at the meeting of the International Dental Congress, and published in the December, 1900, *Dental Cosmos.*

The author cites the well-known theories as to the process by which the teeth emerge from the gums, and offers some criticisms on their adequacy. These are: The elongation of the roots, and the result of masticating force on the alveolar process.—*Pierce.* The gradual contraction of the alveolar sockets.—*Tomes.* The lifting power of the socket attached to the gum above and the neck of the tooth below.—*Delabarre.* The interstitial development of bone in the jaws and process.—*Coleman* A deposit of bone in the bottom of the crypts in which the teeth are developed, and lastly by the gradual consolidation and contraction of the alveolus. The author then gives reasons why these theories will not fully account for the phenomenon, and states his theory in the following words: "*The blood pressure exerted in the vascular tissue which lies between the developing tooth and its bony surroundings is the active mechanical factor in the process.*"

In the jaws of a nine months' fetus the partially calcified cusps of the temporary teeth are imbedded in soft, gelatinous material which is inclosed in bony crypts. The calcified portions of the developing teeth are not at any point in contact with the walls of the crypts in which they are severally contained, but on every side the gelatinous material intervenes. Later on, as calcification proceeds, the cusps more nearly approach the walls of the crypts—which latter do not appear to undergo any enlargement. When the crowns of the developing teeth are fully calcified, the walls of the crypts have undergone partial absorption, the bone covering the cusps of the molars and the labial surfaces of the incisors being removed. Even at this time there is no actual contact between the crowns and the remaining walls of the crypts, the nearest approach to contact being around the sides of the crowns. Between the bases of the crowns and the floors of the crypts there exists a varying but always easily appreciable space filled in with soft vascular tissue, which extends up between the sides of the crowns and the walls of the crypts.

From this time onward, coincident with root-formation, elevation of the tooth crown takes place, and what was the wall of the crypt becomes the socket of the root; but between it and the forming root is the same vascular tissue that separated the calcifying crown from the floor of the crypt. After the tooth crown has emerged from the gum, and before the root is quite complete, the tissue between the upper part of the root and what we should now term the alveolar wall—viz., the crypt wall which has grown up with and around the tooth—loses its gelatinous character, becoming somewhat tougher and more adherent to the root and to the alveolar wall; but it maintains its gelatinous character between the base of the root and the floor of the alveolus. After the root is fully formed the whole of the gelatinous material on the outside of the root assumes the familiar appearance of the peridental membrane.

In all essential points the process of eruption of the permanent corresponds with that of the temporary teeth. The physiological process of absorption which assists in the removal of the temporary teeth is probably identical with that which removes part of the crypt wall to admit of the elevation of the crowns of the temporary teeth.

Dissection of the jaws of the lamb and the pig evidences, from the similarity in the anatomical disposition of the developing teeth and the adjacent parts, that the mechanism of eruption is the same in the case of those animals as in the human subject. The only observable difference is that the gelatinous material is present in greater quantity.

In the case of animals with teeth growing from persistent pulps, it is important to note that the anatomical relations of such teeth are precisely similar to those of human teeth with partially developed roots. In other words, a tooth growing from a persistent pulp has its root imbedded in gelatinous material, and is nowhere in contact with its bony surroundings. It is fair, therefore, to assume that the force which thrusts forward the tooth with a persistent pulp is identical with that causing the eruption of a developing human tooth. From the foregoing description it is evident that the only one of the theories previously enunciated which has any semblance of anatomical foundation is that which states that the eruption of the teeth is due to the elongation of their roots. There is no evidence of "bone currents," of a contractile sac such as assumed by Delabarre, or of a deposit of bone at the bottom of the crypt.

It appears to the writer that the chief objection to the root-elongation and bone-formation theories is a physiological one. It is extremely difficult to conceive such a process as dentin formation exercising independent mechanical force! But, granting that it may be so, upon what structure is that force exercised? In other words, to put the matter clearly and concisely, if somewhat vulgarly, what does the root shove against?

Since the forming root is never in actual contact with its bony surroundings, it must necessarily be against the vascular material in which it is imbedded. Now, this tissue appears *post-mortem* of far too jelly-like a consistence to oppose any effective resistance by virtue of its own structure, and yet such resistance there must be or the tissue would be obliterated. Whence, then, are its resisting properties derived? Necessarily from the blood pressure. Therefore, assuming that the physiological process of dentinification *can* exercise independent mechanical force and *is* a factor in the causation of eruption, it must, since action and reaction are equal and opposite, divide the honors with the blood pressure, a factor hitherto quite unrecognized. Indeed, it is obvious that any *vis a tergo* must act through the vascular material surrounding the root, and it follows that such force can not be greater than the blood pressure or it would cut off the blood supply to the root.

But is a force other than the blood pressure a necessary hypothesis when we consider the exceptionally advantageous conditions under which it acts?

The calcified crown of a molar tooth covered above by the soft tissues of the jaw, but otherwise surrounded by bony walls, from which it is separated by a soft and gelatinous but extremely vascular tissue, which tissue is injected by the force of the blood pressure, entering almost directly from an artery of considerable size.

Immediately adjoining is a fully erupted molar with its roots imbedded in a tissue, which is extremely vascular but is tougher, also inclosed by bony walls.

Now, it is obvious that the blood pressure exerted in the gelatinous tissue acts upon its tooth crown at a considerable mechanical advantage. First, from the fact that the tissue is inclosed by the unyielding walls of the crypt, and, secondly, from the embryonic character of the tissue itself. In fact, with regard to this point, it is a marvel that dentists, who have many opportunities of observing the rapidity with which teeth sometimes move during eruption, should ever have been induced to regard the comparatively slow process of dentinification as the active agent in the matter.

We find in the case of the fully erupted molar that the space between the roots and the socket is occupied by the peridental membrane, which forms a ligament for the tooth, being attached both to the roots and to the socket. The vascularity of this membrane still endows it, however, with an extrusive tendency, which is in itself sufficient to account for the gradual elongation ot unopposed teeth, and is probably the chief means by which the proper occlusion of opposing teeth is maintained. If we have any doubts in the case of healthy teeth as to whether this elongation is due to the normal blood pressure exerted in the peridental membrane, they should surely be dispelled by the clinical phenomena that present themselves when the blood pressure is pathologically augmented; in other words, when inflammation of the peridental membrane supervenes.

If we assume that the blood pressure, acting in the manner above described, is the sole active mechanical factor in determining the eruption of the teeth, will it account for the phenomena other theories fail to explain? That the crown of a tooth sometimes travels a distance greater than the length of its root; that teeth sometimes erupt subsequently to the formation of their roots, and that teeth with comparatively little roots occasionally erupt, are facts all in favor of such hypothesis, and can be explained by it.

The fact that teeth with fully formed roots remain unerupted can be more easily explained by this theory than by any other, because it alone can account for the space obtained for the fully developed roots which often occupy abnormal positions. In other words, the blood pressure, acting as it does equally in all directions, makes room for the developing root in the direction of least resistance. Normally, this is in the direction of the advancing crown, but occasionally it is elsewhere.

The continuous eruption of teeth with persistent pulps, which neither Delabarre's nor Coleman's theory could possibly explain, is a very simple problem if we admit the blood pressure as the active mechanical factor.

The writer is therefore of opinion that upon anatomical and physiological grounds alone we are justified in assuming that *the blood pressure exerted in the vascular tissue which lies between a developing tooth and its bony surroundings is the active meehanical factor in the process known as the eruption of teeth.*

So far the purely mechanical aspect of the question has been alone considered, but if your patience will endure the strain, there are two points of physiological interest that have so direct a bearing upon the subject that it would be as well to include them here.

Some years ago the writer recorded the observation that in young people, when a back tooth had lost its antagonists, the characteristic elongation which takes place under those circumstances varied in cases in which the pulps of the unopposed teeth were dead from those in which they were living. In the former instance, although the elongation of the teeth took place, it was unaccompanied by any downgrowth (or, in the case of a lower tooth, upgrowth) of the alveolar ridge; whereas in the latter case there *was* a corresponding deepening of the alveolar ridge. In other words, in the case of dead teeth there was simply extrusion from the alveolus, but in the case of living teeth there was growth of the alveolar ridge. It appears obvious, therefore, that the growth of the alveolar process is dependent upon the integrity of the dental pulp, or, in other words, that the pulps of the teeth as a whole exercise a trophic influence with regard to the alveolar process. It is the opinion of the writer that extirpation of the pulp of a tooth causes a marked and permanent alteration in the vascular condition of the peridental membrane ; in fact, a disturbance of vaso-motor equilibrium in the direction of a paralysis of the vaso-constrictor mechanism.

The foregoing remarks apply to both the permanent and temporary dentitions. But the pulps of the temporary teeth exercise another kind of trophic influence which seems to have escaped the notice of dental writers —namely, their influence upon the process of resorption of the roots of the temporary teeth. In the case of temporary teeth, in which the pulps are destroyed at the time when resorption of the roots should commence, resorption, strictly so-called, does not occur at all. In such cases a certain amount of absorption of the root, as a rule, takes place, just as it often does in the case of dead permanent teeth, the microscopic appearance of the roots in both cases being strikingly similar. This absorption is a pathological process, and differs markedly from the physiological process of resorption. It is a much slower process, and that is one reason why we so frequently find the apices of the roots of dead temporary teeth protruding from the labial or buccal surfaces of the alveolar ridges, causing that ulceration of the mucous membrane of the cheeks or lips with which we are so familiar in the case of children whose milk teeth have been neglected.

The explanation of this common phenomenon is simple. The death of the pulp of the temporary tooth has left its root incable of resorption, and its socket prone to degeneration. Absorption is too slow a process to make room for the crown of the permanent successor, which soon impinges upon the dead root, deflects it, and thrusts its apex through the degenerated alveolar process and the superjacent soft tissues. In those cases in which death of the pulp of the temporary tooth has taken place some time after the process of resorption has commenced and the root is in consequence shortened, the pressure of the advancing permanent tooth simply tilts the root until it takes a nearly horizontal position, the crown, if any remain, being correspondingly deflected. Other phenomena which admit of a similar explanation are familiar to us all, and need not be enumerated.

From the time when the dental pulp is "nothing more than a part of the mesoblastic myxomatous tissue of the jaw, which has become more rich in vessels and cells than the other neighboring part" (for this euphonious definition the writer is indebted to his distinguished countryman, Mr. Tomes, "Dental Anatomy," page 129), up to the time when commencing senile degeneration presages the termination of its physiological activity, it is one of the busiest exponents of local government observable in the whole domain of human physiology. While it is hard at work constructing the tooth it regulates the blood pressure that causes that organ to travel to its appointed place in the mouth, at the same time building up the bony walls that enable that pressure to act at a mechanical advantage. Then, in the case of the temporary teeth, it superintends the demolition of the very structure it has been at such pains to create; and finally, in the case of the permanent teeth, it controls the nutrition of those parts upon the integrity of which the tooth is dependent for the proper exercise of its function. Thus we are able to understand the otherwise inexplicable phenomenon which Mr. Tomes aptly describes in the following words: "It is impossible to insist too strongly upon the fact that the sockets grow up with and are moulded around the teeth as the latter elongate. Teeth do not come down and take possession of sockets more or less ready-made and pre-existent, but the socket is subservient to the position of the tooth. Wherever the tooth may chance to get to, there its socket will be built up round it. Upon the proper appreciation of this fact depends our whole understanding of the mechanism of teething. The position of the teeth determines that of the sockets, and the form of pre-existent alveolar bone has little or nothing to do with the disposition of the teeth."

